# Comparison of Immunohistochemistry Expression of CK7, HMWK and PSA in High-Grade Prostatic Adenocarcinoma and Bladder Transitional Cell Carcinoma

**DOI:** 10.30699/ijp.2020.123998.2353

**Published:** 2020-11-13

**Authors:** Reza Gheitasi, Esmaeil Sadeghi, Mohammad Jafari

**Affiliations:** 1 *Institute for Infectious Diseases and Infection Control, Jena University Hospital, Jena, Germany *; 2 *Department of Immunology, School of Medicine, Hamadan University of Medical Sciences, Hamadan, Iran *; 3 *Department of Medicine, School of Medicine, Hamadan University of Medical Sciences, Hamadan, Iran*; 4 *Department of Pathology, School of Medicine, Hamadan University of Medical Sciences, Hamadan, Iran*

**Keywords:** Prostate Adenocarcinoma, Cytokeratin 7, High-Molecular-Weight Cytokeratin, Prostate-Specific Antigen Urothelial Bladder Carcinoma

## Abstract

**Background & Objective:**

Prostate adenocarcinoma is the most common malignancy in males, and the urothelial bladder carcinoma is also prevalent. The histological characteristic of these two tumors is very similar in high-grade cases, and their differentiation is difficult. This study was performed to compare the immunohistochemistry panel of high-grade prostate adenocarcinomas and high-grade urothelial bladder carcinomas.

**Methods:**

In this cross-sectional study, 36 cases of prostate adenocarcinoma and 36 urothelial bladder carcinoma samples were collected from the pathology department of Shahid Beheshti Hospital in Hamedan. For each sample, expression of Cytokeratin 7, high-molecular-weight cytokeratin and Prostate-specific antigen markers was evaluated by immunohistochemistry. Comparison of expression of these markers in high-grade bladder tumors and prostate tumors was made by SPSS 25 using Chi-square test.

**Results:**

In this study, the Cytokeratin 7 positivity was seen in 88.9% of bladder cancer versus 27.8% of prostate cancer samples. High-molecular-weight cytokeratin positive immunoreactivity was noted in 55.6% of bladder cancer and 5.6% of prostate cancer samples. Prostate-specific antigen marker showed positive results in 94.4% of prostate cancer samples, but no positivity was evident in those of bladder cancer.

**Conclusion:**

A panel of immunohistochemical stains can be used to differentiate high-grade prostate adenocarcinoma from urothelial bladder carcinoma in those cases which are challenging to diagnose.

## Introduction

Prostate cancer (PCa) is the most common malignancy in men in the United States which constitutes 29% of all cancer (1). It is the second leading cause of death due to malignancy after lung cancer (2). The prostate adenocarcinoma accounts for more than 90% of the epithelial malignancies of this organ (3). Rectal examination is a practical and useful way to diagnose PCa (due to the posterior position of most tumors), but it has low sensitivity, specificity, and pathologic confirmation is always needed, however, in the early stages, the carcinoma is not differentiated from lesions such as nodular hyperplasia, granulomatous prostatitis, tuberculosis, prostate infarction, or stones (4, 5). Rectal ultrasonography can detect small 5 mm carcinomas that are a hypoechoic lesion. However, this method does not diagnose 30% of prostate tumors that are iso-echo and is not an excellent tool for screening (6, 7). High-grade prostate carcinomas and bladder tumor overlap regarding clinical manifestations and morphological characteristics, which is why sometimes it is not possible to distinguish them in an optical microscope (8), and we need to use other possibilities to differentiate them. One of the most suitable methods for differentiation is the use of immunohistochemistry (IHC) because many immunohistochemistry markers are different in these two tumors (9, 10). Prostate-specific antigen (PSA) is an appropriate marker for PCa, and Cytokeratin 7 (CK7) and high-molecular-weight cytokeratin (HMWK) clone‎ ‎34β£12‎‏ ‏ are suitable markers for bladder carcinoma (11), and their combination can play a significant role in differentiating these cancers (12, 13). Two prostatic epithelium Immunohistochemical markers that can be demonstrated in samples that have been processed with polyclonal or monoclonal anti-serum are prostatic acid phosphatase (PAP) and PSA (14). These two markers usually have been used to approve a prostatic tumor origin (15); however, they are not expressed uniformly in poorly differentiated Prostatic Cancer and might be negative in up to 27% for PSA and 19% of cases for PAP (16). They do not differentiate benign and malignant prostate cancer, but they are used to diagnose the origin of metastatic tumors. These markers are useful in ‎the diagnosis of prostate tumors which slightly differentiated, and transitional bladder tumors (17). Several studies have reported that PSA is more intense, more stainable and more specific than PAP, especially when monoclonal antibodies are used (18-20). However, this specificity is not absolute because the immunoreactivity resembles PSA is also observed in normal salivary glands, neoplasms, some breast carcinomas, and several human tissues (21). Due to the specificity of PSA for prostate gland epithelium, this marker is very useful for the diagnosis of prostate adenocarcinoma in adjacent tissues such as rectum and bladder. Rectum adenocarcinoma, urothelial carcinoma, or bladder adenocarcinoma cannot produce PSA (22). The ‎34β£12‎ or HMWK antibody is a non-sensitive but highly specific marker for differentiating the transitional bladder carcinoma from prostate adenocarcinoma (11, 23). The ‎34β£12‎ antibody specifically detects the basal cells. This antibody is useful in differentiating the prostate adenocarcinoma and benign lesions of the organ. The ‎34β£12‎ antibody detects HMWK present in the basal cells of the prostate gland and therefore has significant diagnostic value (24). This marker is consistently present (Sometimes non-continuous) in benign glands and does not exist in adenocarcinoma, regardless of the grade (25). Cytokeratins are a group of water-soluble fiber proteins that appear in the most epithelium (26). The occurrence of creatine can be a useful marker for the diagnosis of epithelial tumors and distinguishes tumors from the origin of endodermal, neuroectodermal, mesenchymal, or germ cell cells (27). Cytokeratin 7 is a basic cytokeratin that is found in most of the glandular and transitional epithelium, but not in the squamous epithelium. CK7 is commonly found in lung, breast, ovary (serous and endometriotic tumors), uterine cervical tumors, biliary epithelium and cholangiocarcinoma and transitional cell carcinoma (TCC), but do not exist in squamous cell carcinomas. The most important differential diagnosis of TCC and prostate adenocarcinoma is an IHC panel which includes Leu7 (human natural killer-1), PSAP, PSA, P63, CK7, and CK34BE12. The first three markers are positive in TCC, and the next three are positive in prostate adenocarcinoma. All together there is challenge in differentiation between UCa and PCa to avoid misdiagnosis. Here we aimed to show a compact immunohistochemistry panel that could be useful for convenient differentiation between UCa and PCa.

##  Materials and Methods

This cross-sectional study was performed on 36 patients with PCa and 36 patients with bladder carcinoma. The participants in this study included those who were diagnosed with bladder carcinoma or prostate adenocarcinoma admitted to the hospital for surgical intervention. For each specimen, the slides, fixed blocks, and related diagnosis were collected. Patients were divided into two groups: high-grade prostate adenocarcinoma and high-grade urothelial bladder carcinoma. Then, for each sample, one or two appropriate paraffin blocks with a top-rated tumor component were selected, and expression of CK7, HMWK and PSA was evaluated by IHC method. In each case, according to the protocol of the IHC kit (DAKO, Carpinteria, CA) the following steps were taken respectively: 1) Cutting and mounting sections on slides coated with suitable tissue adhesive. 2) De-paraffinizing sections in xylene substitutes. 3) Re-hydrating through graded alcohols. 4) Washing slides in running tap water. 5) Performing antigen retrieval as required. 6) Washing slides in de-ionized water. 7) Neutralizing endogenous peroxidase using peroxidase block for 5 minutes. 8) Washing in TBS for 2 × 5 minutes. 9) Incubating with protein block for 5 minutes. 10) Washing with optimally ready to use primary antibody. 11) Washing in TBS for 2 × 5 minutes. 12) Incubating with post-primary for 30 minutes. 13) Washing in TBS for 2 × 5 minutes. 14) Incubating with linked-vision polymer for 30 minutes. 15) Washing in TBS for 2 × 5 minutes with gentle rocking. 16) Developing peroxidase activity with DAB working solution for 5 minutes. 17) Rinsing slides in water. 18) Counterstain with hematoxylin. 19) Rinsing slides in water for 5 minutes. 20) Dehydrating, clearing and mounting sections. Then, a pathologist and an assistant examined slides by optical microscope and divided them into positive or negative groups for CK7, HMWK, and PSA. Then, to compare markers expression between two groups of patients, Chi-squared test was performed. SPSS version 25.0 (SPSS Inc., Chicago, IL, USA), was used for all statistical analyses. P-value<0.05 was considered as a significant level.

## Results

**Fig. 1:**
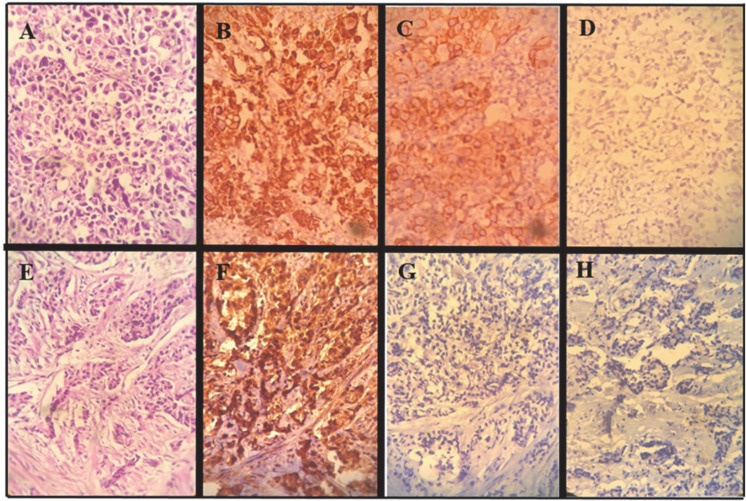
A-D, poorly differentiated transitional cell carcinoma (A, H&E×400), CK7 (B×400), high ‎molecular-weight cytokeratin clone 34βE12 (C×400) and PSA (D×400). E-H, poorly ‎differentiated prostatic carcinoma (E, H&E×400), PSA (F×400), CK7 (G×400) and high-‎molecular-weight cytokeratin clone 34βE12 (H×400).

Patients were divided into two groups, and after ensuring the high grade of the tumor, the expression of CK7, HMWK, and PSA biomarkers were evaluated for all patients (Figure 1). 

The CK7 was positive in 32 urothelial bladder carcinoma patients (88.9%) νs in 10 prostate adenocarcinoma patients (27.8%) (Table 1).

The HMWK was positive in 20 patients (55.6%) with urothelial bladder carcinoma and only in two patient (5.6%) with prostate adenocarcinoma (Table 2).

**Table 1:** Distribution of CK7 by Tumor Type

	CK7		
	Yes	No	
**36** **(100%)**	32 (88.9%)*	4 (11.1%)	**1.Bladder group** **Number** **(% in group)**
**36** **(100%)**	10 (27.8%)	26 (72.2%)*	**2.Prostate group** **Number** **(% in group)**
**72** **(100%)**	42 (58.3%)	30 (41.7%)	**Total** **Number** **(% in group)**

* P-value˂0.05

**Table 2:** Distribution of HMWK by tumor type

	HMWK		
	Yes	No	
**36** **(100.0%)**	20 (55.6%)	16 (44.4%)	**1.Bladder group** **Number** **(% in group)**
**36** **(100%)**	2 (5.6%)	34 (94.4%)*	**2.Prostate group** **Number** **(% in group)**
**72** **(100%)**	22 (30.6%)	50 (69.4%)	**Total** **Number** **(% in group)**

* P-value˂0.05

PSA had positive result in 34 patients (94.4%) with prostate adenocarcinoma but there was no positive result in any of the cases of urothelial bladder carcinoma (Table 3).

**Fig. 2.**
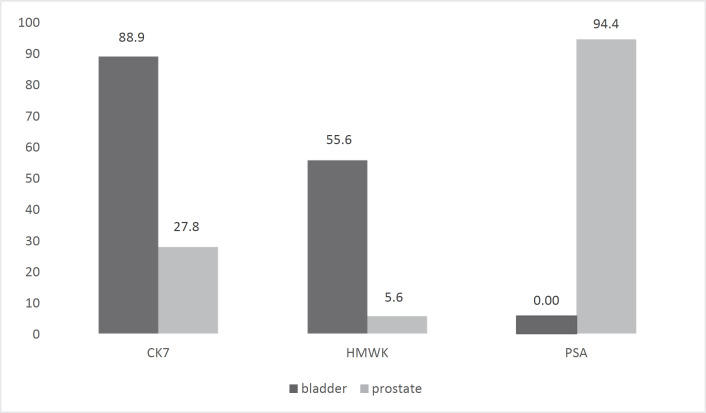
Comparison of CK7, HMWK, and PSA expression in Prostate and bladder tumor

Comparison of CK7, HMWK, and PSA expression in urothelial bladder carcinomas‎ and prostate adenocarcinomas (high-grade) is presented in Figure 2.

**Table 3:** Distribution of PSA by tumor type

	PSA		
	Yes	No	
36 (100.0%)	0 (0.00%)	36 (100%)*	**1.Bladder group** **Number** **(% in group)**
36 (100%)	34 (94.4%)*	2 (5.6%)	**2.Prostate group** **Number** **(% in group)**
72 (100%)	34 (47.2%)	38 (52.7%)	**Total** **Number** **(% in group)**

* P-value˂0.05

## Discussion

Prostate adenocarcinoma is the most common malignant tumor in men, and also bladder cancers are relatively common. As previously mentioned, high-grade of these two tumors are histologically very similar, and sometimes their differentiation is impossible. However, the treatment of these two tumors are different, and also their survival. Therefore, it is important to try to identify how these tumors can be differentiated. We must have definite criteria for the histological differentiation of these two tumors. Therefore, we decided to use the panel to differentiate PCa and bladder transitional cell carcinoma. In this study, 88.9% of bladder tumors and 27.8% of PCa had positive results for CK7. HMWK was positive in 55.6% bladder tumors νs only 5.6% prostate tumors. Moreover, PSA, was positive in 94.4%, of prostate tumors and was not positive in any of the bladder tumors.

In the study by Elizabeth M. genega* et al.* For distinguishing moderate to weak prostate adenocarcinoma and bladder transitional cell carcinoma, they used a large panel of biomarkers including CK_7_, CK_20_, HMWK, leuM_1_, CEA, PSA, Leu_7_ and B_7203_. In this study, PSA was positive in 94% of PCa but was not positive in any of the bladder cancers. More than a half (65%) of the urothelial bladder‎ cancers had positive results for HMWK, while 6% of PCa samples had positive findings. The CK7 was positive in 83% of urothelial bladder cancer‎ and 12% of PCa. They finally expressed that six antibodies including PSA, PSAP, 34Βe12, Leu7, CK7, and P53 are appropriate for this purpose. The first three in the prostate carcinomas and the following third antibodies in the bladder carcinomas are positive. The 34βE12 or HMWK antibody is a non-sensitive but highly specific marker for differentiating the bladder transitional cell carcinoma from prostate adenocarcinoma (23). 

Nader H. Bassily* et al.*‎ in a study on the incidence of two markers, CK7 and CK20, in prostate adenocarcinoma and bladder transitional cell carcinoma, found that the combination of these two markers is useful for differentiating these tumors (28).

In a study performed at William Beaumont Hospital in the United States, it was found that CK7 is positive in 48% of urothelial bladder carcinoma, interestingly, about prostate adenocarcinoma, by increasing Gleason's score in the tumor, the marker's percentage of positivity increased (29). 

In a study by Jesse K. Mckenney regarding the role of IHC in the diagnosis of bladder neoplasms, the incidence of various markers in bladder and prostate cancers was investigated that PSA in prostate cancer was positive in about 68-94%, but there was no positive in any of the cases of bladder cancer. HMWK was reported to be positive in prostate cancer of 10-6%, while this marker was ‎reported to be positive in bladder cancer of 65-100% (30).

Another by Ong C-AJ *et al*,. that they studied the differentiation of high-grade prostatic carcinomas and urothelial bladder carcinoma. PSA had positive results in 97.4% of prostate tumors. However, a remarkable point in these studies was that HMWK was positive in 91.4% of bladder tumors, which was much higher than in other studies, including our studies (55.6%) (31).

The study by Yang* et al.* noted that lack of basal cell markers, such as HMWK, can be helpful in detecting PCa. They evaluated 100 cases of PCa metastases to different locations and observed that only 4 cases of HMWK were positive which two of them are also very poorly stained. They believed that PCa and even high-grade types of it only rarely express HMWK and this marker will remain useful to detect PCa (32).

Shah Rajal* et al.* conducted a study on basal cell markers, including p63 and HMWK, and concluded that lack of staining for these markers is strongly in the benefit of prostate carcinoma (33).

Varma* et al.* used an HMWK to distinguish invasive and high-grade prostate and bladder carcinomas. In this study, there were 20 cases in each group. HMWK was positive in all cases of invasive bladder carcinoma. They expressed that use of HMWK, especially when used with microwave heat retrieval, is a highly sensitive marker for the diagnosis of urothelial carcinoma (34).

Lakshmi* et al.* used the IHC panel to distinguish poorly differentiated prostate from urothelial carcinoma in 36 cases of poorly differentiated UCa and 42 cases of PCa. This panel includes PSA, HMWK (34βE12), CK7, CK20, P63, and α-methylacyle- coenzyme A racemase. They reported that PSA was positive in 95% of PCa and 0% of UCa cases. HMWCK was positive 97% of UCa vs 2% of PCa cases. CK7/CK20 co-expression was stained in 50% of UCa cases; whereas, 14% of PCa cases were positive with both. Finally, they suggested that positive HMWCK with negative PSA establishes the diagnosis of UCa (35). This point was coinciding with our study.

## Conclusion

The Studies mentioned, achieved similar results like our experiment which can be a proof of our achievement. Therefore, we described an immunohistochemical panel of some certain markers used in our study to differentiate high-grade prostate adenocarcinoma from urothelial bladder carcinoma in cases which are challenging to diagnose. We concluded IHC staining for CK7, HMWK, and PSA can be used as a new panel for distinguishing high-grade cases of prostate origin from bladder cancers.
